# Cloning, purification, and characterization of GH3 *β*-glucosidase, MtBgl85, from *Microbulbifer thermotolerans* DAU221

**DOI:** 10.7717/peerj.7106

**Published:** 2019-07-22

**Authors:** Hyo-Min Pyeon, Yong-Suk Lee, Yong-Lark Choi

**Affiliations:** Department of Biotechnology, Dong-A University, Busan, South Korea

**Keywords:** Glycoside hydrolase family 3, Beta-glucosidase, Microbubifer thermotolerans, DAU221, Cellulose, Gene cloning, Organic solvent tolerance

## Abstract

**Background:**

*β*-Glucosidases have attracted considerable attention due to their important roles in various biotechnological processes such as cellulose degradation to make energy and hydrolysis of isoflavone. *Microbulbifer thermotolerans* (*M. thermotolerans*) is isolated from deep-sea sediment and has not been researched much yet. As a potential candidate for a variety of biotechnological industries, *β*-glucosidases from the novel bacterial species should be researched extensively.

**Methods:**

*β*-Glucosidase, MtBgl85, from *M. thermotolerans* DAU221 was purified by His-tag affinity chromatography and confirmed by SDS-PAGE and zymogram. Its biochemical and physiological properties, such as effects of temperature, pH, metal ions, and organic solvents, substrate specificity, and isoflavone hydrolysis, were investigated.

**Results:**

*M. thermotolerans* DAU221 showed *β*-glucosidase activity in a marine broth plate containing 0.1% esculin and 0.25% ammonium iron (III) citrate. The *β*-glucosidase gene, *mtbgl85*, was isolated from the whole genome sequence of *M. thermotolerans* DAU221. The *β*-glucosidase gene was 2,319 bp and encoded 772 amino acids. The deduced amino acid sequence had a 43% identity with OaBGL84 from *Olleya aquimaris* and 35% and 32% identity with to CfBgl3A and CfBgl3C from *Cellulomonas fimi* among bacterial glycosyl hydrolase family 3, respectively. The optimal temperature of MtBgl85 was 50 °C and the optimum pH was 7.0. MtBgl85 activity was strongly reduced in the presence of Hg^2+^ and Cu^2+^ ions. As a result of measuring the activity at various concentrations of NaCl, it was confirmed that the activity was maintained up to the concentration of 1 M, but gradually decreased with increasing concentration. MtBgl85 showed higher enzyme stability at non-polar solvents (high Log *P_ow_*) than polar solvents (low Log *P_ow_*). The hydrolyzed products of isoflavone glycosides and arbutin were analyzed by HPLC.

## Introduction

Cellulose is the most abundant organic biomass in which D-glucose is linked in a straight chain form with *β*-1,4-glycosidic bond (Klemm et al., 2005). Biomass can be used as an energy source by direct combustion, methane fermentation, and alcohol fermentation. Using biomass as an energy source can be used to store and regenerate energy, and can be obtained from anywhere in the world with the appropriate water and temperature conditions (Brosowski et al., 2016).

At least three kinds of degrading enzymes are required to decompose cellulose (Karnaouri et al., 2013). One is endo- *β*-1,4-glucanase (EC 3.2.1.4), which randomly cuts cellulose chains producing glucose and cellooligosaccharides. Cellobiosidase (EC 3.2.1.91) exolytically attacks the reducing or non-reducing end of cellulose for yielding cellobiose, and *β*-glucosidase (EC 3.2.1.21) hydrolyzes from cellobiose or cellooligosaccharides to glucose (Henrissat, 1991; Huang & Forsberg, 1988; Rosales-Calderon, Trajano & Duff, 2014). *β*-Glucosidase constitutes a major group among glycoside-hydrolyzing enzymes belonging to family 1 (GH1) and family 3 (GH3), which catalyze the selective cleavage of glycosidic bonds (Henrissat, 1991; Hong, Tamaki & Kumagai, 2007). Most bacteria, archaea, plant, and animal *β*-glucosidase belong to GH1, whereas some bacteria and all yeast and mold enzymes belong to GH3 (Hwang, Lee & Choi, 2018; Ng et al., 2010).

Among the cellulose degrading enzymes, *β*-glucosidase can convert isoflavones into aglycones. Isoflavones have a similar structure to estrogen and are present in glycoside form (genistin and daidzin) and aglycone form (genistein and daidzein), which are found in soybeans (Hwang, Lee & Choi, 2018). Most isoflavones have various effects such as cancer prevention, osteoporosis, and cardiovascular disease. This effect is due to the aglycone form, not the glycoside form. The high biological activity of isoflavone aglycones is absorbed into the intestine without interruption (Park, Lee & Choi, 2013; Li et al., 2012; Hwang, Lee & Choi, 2018). The hydroquinone glucoside arbutin is a plant derived compound medically applied for to its antiseptic activity. It also has skin whitening properties and thus is widely used in dermatology and cosmetology (Ewa et al., 2017).

*M. thermotolerans*, belongs to gamma-proteobacteria, was isolated from Suruga Bay sediment samples in Japan (Miyazaki et al., 2008). So far, only biophysical properties of some enzymes such as agarase (Takagi et al., 2015), amylase (Lee, Park & Choi, 2015), carbohydrate esterase (Lee et al., 2014), carrageenase (Hatada et al., 2011), chitinase (Lee, Lee & Choi, 2018), and esterase (Lee, 2016) have been researched. In our previous report, *M. thermotolerans* DAU221, a bacterium capable of degrading cellulose, was isolated from the east coast of Korea (Lee et al., 2014). Another study determined the whole genome sequence of *M. thermotolerans* DAU221 (Lee & Choi, 2016). In this study, the novel *β*-glucosidase from *M. thermotolerans* DAU221 was cloned, expressed, and purified. The *β*-glucosidase is the first enzyme studied in *M. thermotolerans* as well as *Microbulbifer* genus. The experimental results reveal several characteristics and indicate that it could be used in the cosmetic, food, medical, and various biotechnological industries.

## Materials and Methods

### Chemicals

*p*-Nitrophenol (*p*NP), *p*-nitrophenyl- *α*-D-glucopyranoside (*p*NP*α*G), *p*-nitrophenyl- *β*-D-glucopyranoside (*p*NP*β*G), *p*-nitrophenyl-*β*-D-cellobioside (*p*NP*β*C), *p*-nitrophenyl- *α*-L-rhamnopyranoside (*p*NP*α*R), *p*-nitrophenyl- *β*-D-xylopyranoside (*p*NP*β*X), *p*-nitrophenyl-*β*-D-galactopyranoside (*p*NP*β*Gal), *o*-nitropheyl-*β*-D-galactopyranoside (*o* NP*β*Gal), 4-methylumbelliferyl- *β*-D-glucopyranoside (MU*β*G), isoflavone glycoside standards (genistin and daidzin), isoflavone aglycone standards (genistein and daidzein), arbutin, avicel, esculin, ammonium iron (III) citrate, cellobiose, maltose, lactose, sucrose, and xylan were obtained from Sigma (St. Louis, MO, USA). All the different chemicals and reagents were used at analytical grade.

### Bacterial strains, plasmids, and media

*M. thermotolerans* DAU221 (KCCM 43021) was isolated from seawater obtained from goraebul beach in Korea (Lee et al., 2014). The plasmid pCold I (TaKaRa, Kyoto, Japan) was used for expression of fusion protein. *Escherichia coli* (*E. coli*) JM109 and BL21 (DE3) were used as the cloning and protein expression hosts, respectively. The hosts were cultured in Luria-Bertani (LB) medium (10 g of polypeptone, 5 g of yeast extract, and 5 g of NaCl in 1 L of deionized water, pH 7.0) containing ampicillin (50 µg/ml) at 37 °C.

### Cloning and amino acid sequence analysis of *mtbgl85* gene

The following oligonucleotides primers were synthesized to amplify the *mtbgl85* gene based on the whole genome sequence of *M. thermotolerans* DAU221: DAU221-BGL2174-SP-F1 (5′-AAGCTTGCCAACGAATCTGTGGCTA-3′) and DAU221-BGL2174-R1 (5′-TCTAGATTATTGCAGGGTAAAGCTGCCC-3′). *Hin* d III and *Xba* I restriction enzyme sites (italics and underlined) were introduced into the primers. The reaction was performed using ExPrime Taq DNA polymerase (GeNetBio, Daejeon, Korea) in a TaKaRa PCR thermal cycler. An amplified PCR product of approximately 2.3 kb was subjected to a double digestion with *Hin*d III and *Xba*I, and then ligated into pCold I/*Hin*d III/*Xba*I (pCold-*mtbgl85*). The recombinant plasmid, pCold-*mtbgl85*, was transformed into *E. coli* JM109 and BL21 (DE3). Sequence analysis of MtBgl85 were performed using BLAST (National Center for Biotechnology Information, NCBI) and the ClustalW program (Thompson, Higgins & Gibson, 1994).

### Overexpression and purification of MtBgl85

The recombinant *E. coli* BL21 (DE3) harboring pCold-*mtbgl85* was grown in LB broth containing 50 µg/mL ampicillin for 3 h at 37 °C (OD_600_ = 0.4–0.5), 0.1 mM isopropyl-*β*-D-thiogalactoside (IPTG) was added to induce the overexpression of MtBgl85 at 15 °C for 24 h. Growing cells were harvested by centrifugation for 20 min at 6,000 rpm and 4 °C. Collected cells were resuspended with His-tag binding buffer: 20 mM sodium phosphate, 0.5 M NaCl, 5 mM imidazole, pH 7.0. The cells were disrupted by sonication. Then, the supernatant was collected by centrifugation at 13,000 rpm for 20 min and 4 °C. The His-Trap HP column (Amersharm Biosciences) was used for purification. The column was equilibrated with His-tag binding buffer. After then, supernatant was loaded onto column. The protein attached to the column was eluted with elution buffer: 20 mM sodium phosphate, 0.5 M NaCl, 0.5 M imidazole, pH 7.0. The identified fractions were collected and desalted by Amicon Ultra-4 (Millipore Bedford, MA, USA).

### SDS-PAGE and zymogram of MtBgl85

The molecular weights of the MtBgl85 were determined by sodium dodecyl sulfate-polyacrylamide gel electrophoresis (SDS-PAGE) (Laemmli, 1970). The gels for SDS-PAGE consisted of separating gel (10%) and stacking gel (5%). Protein molecular size marker (Elpisbio, Daejeon, Korea) was used as a reference. After SDS-PAGE, the gel was stained with 0.05% Coomassie brilliant blue R-250 for 1 h. The gel was then decolorized using destaining solution (methanol: water: glacial acetic acid = 5: 4: 1) overnight. To view the enzyme activity in the gel, the enzyme was loaded onto the polyacrylamide gel containing 0.1% esculin and 0.25% ammonium iron (III) citrate or Mu *β*G, as the substrate, respectively (Cao et al., 2015; Chamoli et al., 2016). After loading, the gel was immersed in refolding buffer (20 mM sodium phosphate buffer [pH 7.0], 1% Triton-X 100) overnight and then incubated at 50 °C for 30 min. A yellow or light brown color was visualized for the gel containing esculin and ammonium iron (III) citrate and a clear zone was visualized for the gel containing MU*β*G under UV_365_.

### Enzyme assay

Enzyme activity of MtBgl85 was assayed using *p*NP*β*G as substrate. For this, 200 µL of reaction mixture containing 1 mM *p*NP*β*G (final concentration) and diluted MtBgl85 in 20 mM sodium phosphate buffer (pH 7.0) was incubated for 15 min at 50 °C. After incubation, 800 µL of 0.5 M Na_2_CO_3_ was added to stop the reaction. The activity was measured at 410 nm. One unit of enzyme activity was defined as the amount of enzyme required to release of 1 µmol of *p*-nitrophenol per minute.

### Characterization of MtBgl85

The optimal temperature of MtBgl85 was measured at different temperatures (10–80 °C). Thermal stability was carried out by preincubating the enzyme for 1 h without *p*NP*β*G at different temperatures (10–80 °C) and then residual activity was measured under the optimal temperature for 15 min.

The optimal pH was measured in different pH buffers: citrate buffer (pH 3.0–6.0), sodium phosphate (pH 6.0–8.0), Tris-HCl (pH 8.0–9.0), and glycine-NaOH (pH 9.0–10.0). The pH stability was carried out by measuring the residual activity at the optimal temperature, after preincubating the enzyme at 4 °C for 1 h in the various pH buffers without *p*NP*β*G.

To determine the effect of the metal ions, the enzyme was preincubated at 4 °C for 1 h with 1, 5, or 10 mM metal ions, respectively. Mg^2+^, Cs^+^, Na^+^, K^+^, Ca^2+^, Li^+^, Co^2+^, Ba^2+^, Mn^2+^, Zn^2+^, Al^2+^, Sr^2+^, Cd^2+^, Cu^2+^, and Hg^2+^ were used to test for the metal ions.

The salinity activity was determined by using various NaCl concentrations (0.25 M, 0.5 M, 0.75 M, 1 M, 1.25 M, 1.5 M, 2 M, and 2.5 M). The enzyme was preincubated at 4 °C with various concentration NaCl for 1 h. Enzyme activity was measured at the optimal condition with *p*NP*β*G as the substrate.

The effect of organic solvents activity was determined by using various solvents (acetic acid, acetone, butanol, DMSO, ethanol, methanol, isoamyl alcohol, isopropyl alcohol, hexane, benzene, toluene, and acetonitrile). The final concentration of organic solvents was 10%, 30%, and 50%. The reaction mixture consisted of 20 mM sodium phosphate buffer (pH 7.0), 1 mM *p*NP*β*G, each organic solvents, and purified enzyme. The assay containing each organic solvents was incubated at 50 °C for 15 min.

### Substrate specificity and kinetics of MtBgl85

To determine the substrate specificity of MtBgl85, the purified enzyme was incubated with 1 mM of *p*-nitrophenyl substrates, including *p*NP*α*G, *p*NP*β*G, *p*NP*β*C, *p*NP*α*R, *p*NP*β*X, *p*NP*β*Gal, *o*Np*β*Gal, and Mu*β*G, and 1% saccharides, such as arbutin, avicel, cellobiose, esculin, lactose, maltose, sucrose, and xylan, at 50 °C for 15 min, respectively. The reducing sugar released was measured using the DNS method.

For estimating the kinetic parameters, the initial rate of enzyme reaction was calculated with *p*NP*β*G at a final substrate concentration of 0.02–0.1 mM based on the Lineweaver-Burk plot using the SWIFT II Application software (Amersham Bioscience).

### Analysis of hydrolysis products

Hydrolysis products were also analyzed by high performance liquid chromatography (HPLC). A Waters 1500 series HPLC system (Waters Technologies Corporation, Milford, MA, USA) equipped with binary pump, autosampler, UV/visible detector, and OptimaPak C_18_ column (250 × 4.6 mm, 5 µm; RStech, Daejeon, Korea) was used. For the analysis of daidzin and genistin, solution A consisted of water with 0.1% acetic acid and solution B consisted of acetonitrile with 0.1% acetic acid. The standards of genistein and daidzein were diluted in ethyl alcohol. Then, 1 µL of the samples injected with solution B were run at 5% for 5 min, increased from 5 to 35% for 15 min, and run at 35% for 20 min at a flow rate of 1 mL/min. The chromatograms were measured at 260 nm (Park, Lee & Choi, 2013; Raimondi et al., 2009). For the analysis of arbutin, mobile phase was water: methanol (9:1, vol:vol). The standards of arbutin and hydroquinone were diluted in water. The chromatogram was measured with isocratic for 15 min at 280 nm (Ewa et al., 2017).

### Nucleotide sequence accession numbers

The nucleotide sequence reported in this study has been deposited in GenBank under the accession number MK408673.

## Results and Discussion

### Amino acid analysis of MtBgl85

**Figure 1:**
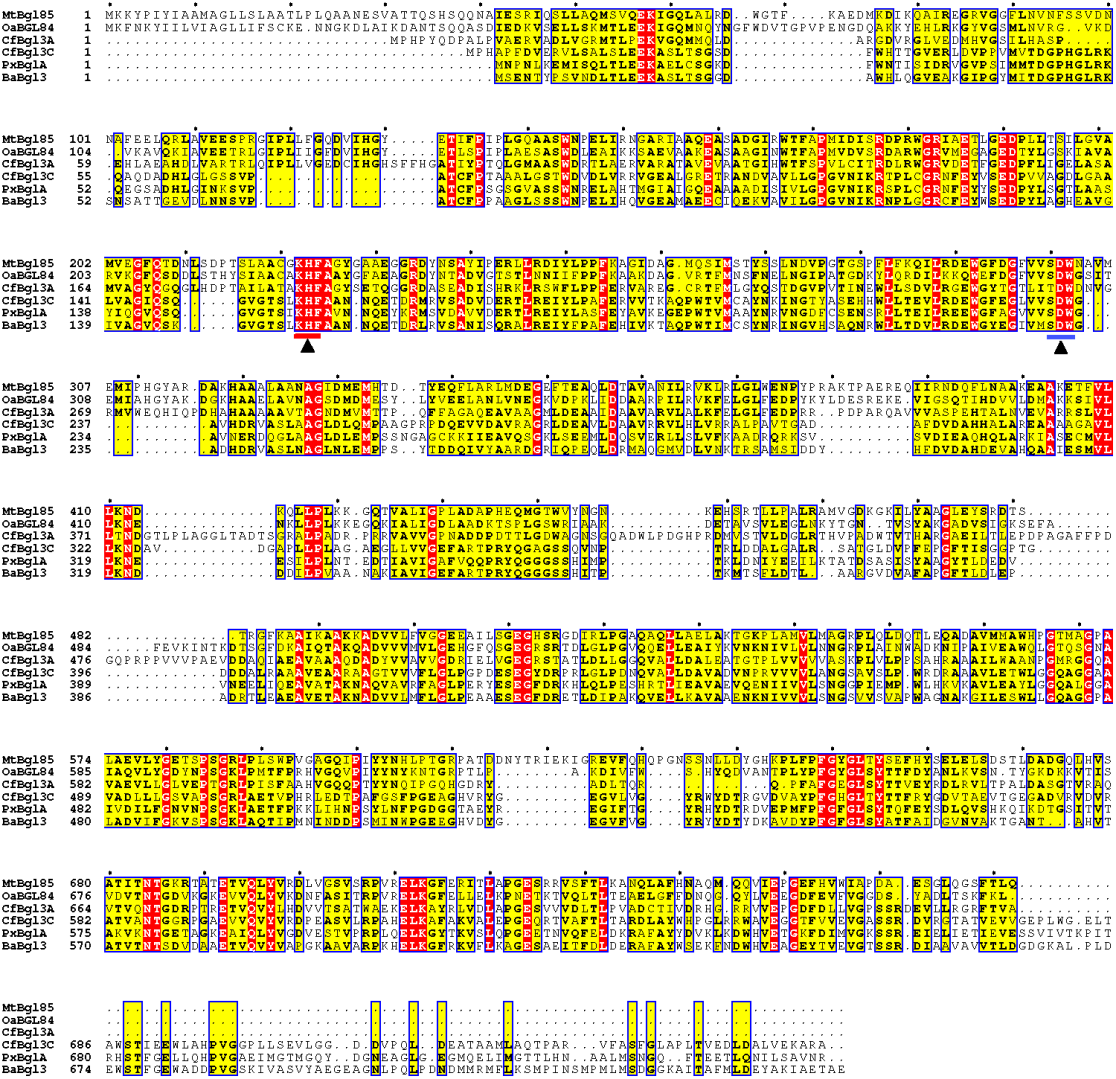
Alignment of MtBgl85 amino acid sequence with other members of bacterial glycoside hydrolase family 3. Similar sequences are marked by boxes and highlighted in yellow and identical sequences are highlighted in red. The KHF and SDW motifs are marked with blue and red lines, respectively. The conserved amino acids are marked with a black triangle. MtBgl85: * β*-glucosidase from *Microbulbifer thermotolerans* DAU221 (MK408673); OaBGL84: * β*-glucosidase from *Olleya aquimaris* DAU311 (KU882052); CfBgl3A (AEE44289) and CfBgl3C (AEE47485): * β*-glucosidases from *Cellulomonas fimi* ATCC 484; PxBglA: * β*-glucosidase from *Paenibacillus xylailyticus* KJ-03 (JN872735); and BaBgl3: * β*-glucosidase from *Bifidobacterium adolescentis* ATCC 15703 (5WAB_A).

**Figure 2:**
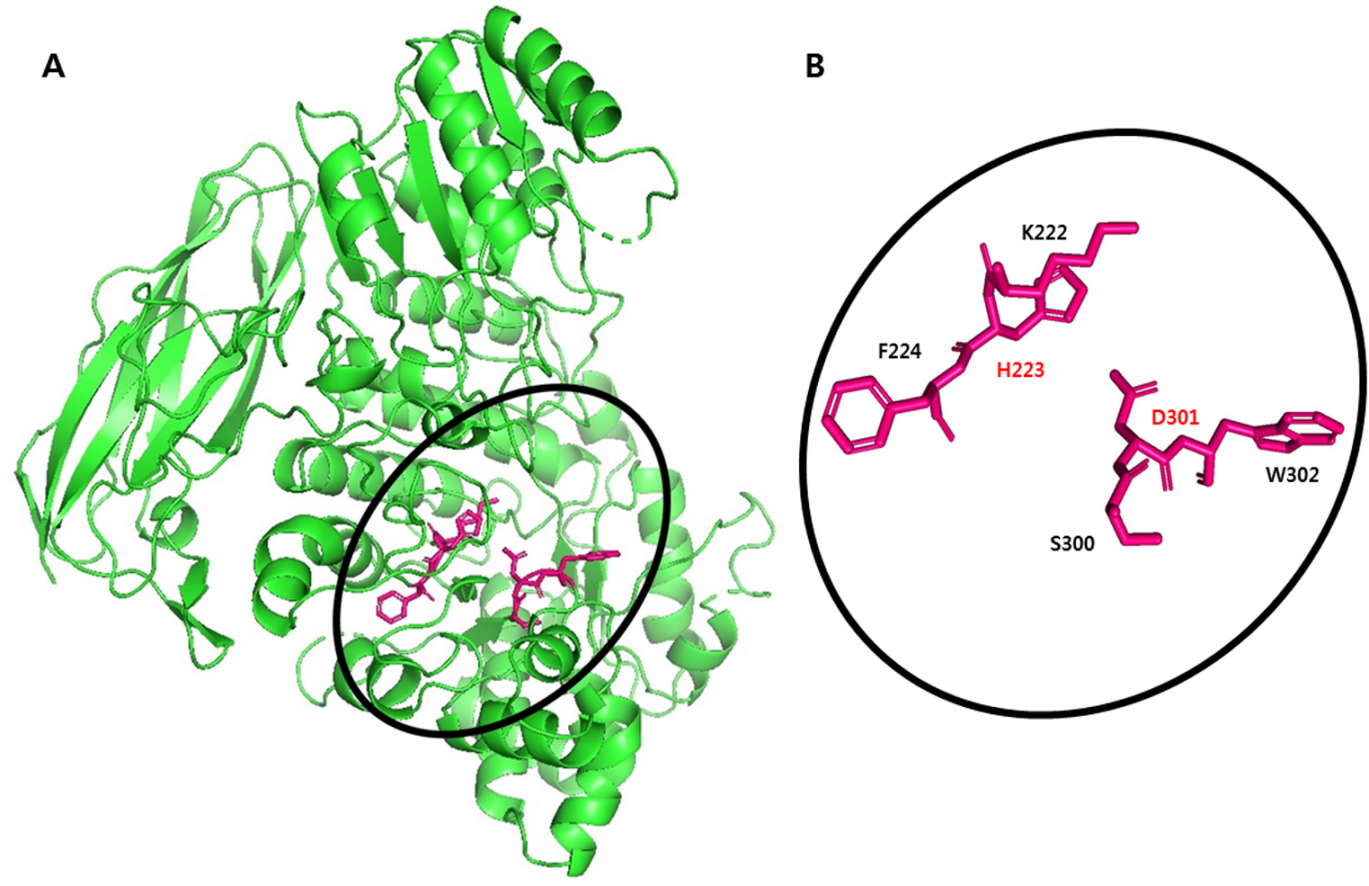
Three-dimensional structural features of MtBgl85. (A) Schematic representation of MtBgl85 model (green). The carbon atoms of the catalytic residues are shown in red. (B) Detailed picture of catalytic residues in the conserved motifs: KHF and SDW.

The *β*-glucosidase gene, *mtbgl85*, was 2,319 bp encoding 772 amino acid, based on the whole genome sequence of *M. thermotolerans* DAU221 (Lee, 2016; Lee & Choi, 2016) (Fig. S1). The signal peptide sequence was found at the *N*-terminus of MtBgl85 of *M. thermotolerans* DAU221 using the SignalP 4.1 server (http://www.cbs.dtu.dk/services/SignalP/) (Nielsen, 2017). The most likely cleavage site is presumed to be between Ala_25_ and Ala_26_. Therefore, the mature protein was predicted to contain 747 amino acids with an estimated molecular mass of 81,924 Da. The signal peptide was removed for cloning and purification. The confirmed amino acid of MtBgl85 was compared with previously known amino acid sequence of bacterial *β*-glucosidases. MtBgl85 showed 43% identities (339/792) and 60% similarities (478/792) with OaBGL84 (KU882052) from *Olleya aquimaris*, 35% identities (272/785) and 49% similarities (392/785) with CfBgl3A (AEE44289) and 32% identities (207/655) and 45% similarities (298/655) with CfBgl3C (AEE47485) from *Cellulomonas fimi*, 30% identities (189/638) and 47% similarities (302/638) with PxBglA (JN872735) from *Paenibacillus xylailyticus*, and 31% identities (197/634) and 46% similarities (297/634) with BaBgl3 (5WAB_A) from *Bifidobacterium adolescentis*. Multiple sequence alignment analysis with other *β*-glucosidase indicated that MtBgl85 of *M. thermotolerans* DAU221 belongs to a typical glycoside hydrolase family 3 (Fig. S2) and maintains the conserved sequence of GH 3 (Fig. 1). The catalytic activity of GH3 depends on aspartic acid (Asp) and glutamic acid/histidine (Glu/His) residues. It has been suggested that the Asp residue acts as a nucleophile and the Glu/His residues act as a proton donor (Bhatia, Mishra & Bisaria, 2005; Hwang, Lee & Choi, 2018; Singhania et al., 2013). MtBgl85 shares the Asp_300_ (D) of the (S/T)DW motif, the active site as the nucleophile, and the KH *F*_(221−223)_ motif, a proton donor (Bhatia, Mishra & Bisaria, 2002; Bhatia, Mishra & Bisaria, 2005) (Fig. 2).

### Cloning and purification of MtBgl85

**Figure 3:**
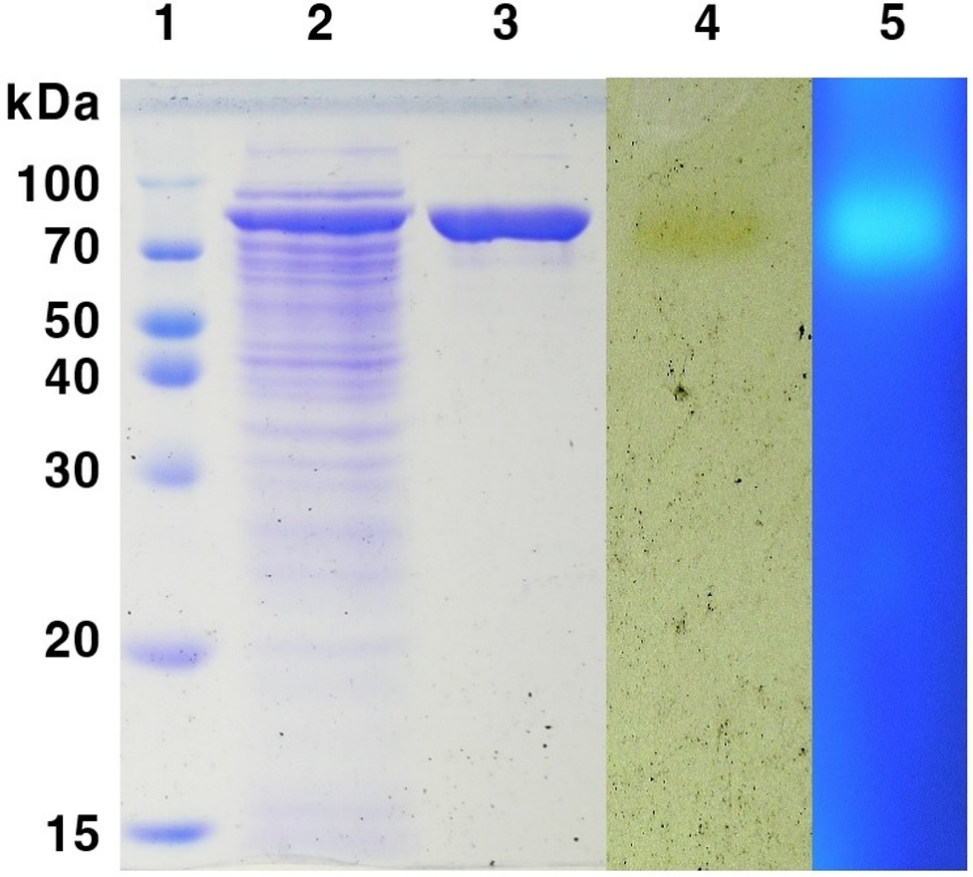
SDS-PAGE analysis of MtBgl85 from *M. thermotolerans* DAU221. Lane 1, protein marker; lane 2, cell free extracts; lane 3, purified MtBgl85 by His-tag affinity chromatography; lane 4, zymogram with 0.1% esculin and 0.25% ammonium iron (III) citrate; lane 5, zymogram with MU*β*G.

The *mtbgl85* gene, except for the signal peptide region, was cloned into the expression vector, pCold I, which allowed for overexpression in *E. coli* BL21 (DE3). The recombinant plasmid, pCold-*mtbgl85*, has 6 histidine tag regions at the *N*-terminal for purification. MtBgl85 was purified by His-tag affinity chromatography. The molecular weight of MtBgl85, predicted to be approximately 85 kDa, was confirmed by SDS-PAGE. Through zymogram analysis, the clear zone was identified around a single band. This results confirmed MtBgl85 is monomeric enzyme (Fig. 3).

### Temperature and pH profiles of MtBgl85

**Figure 4:**
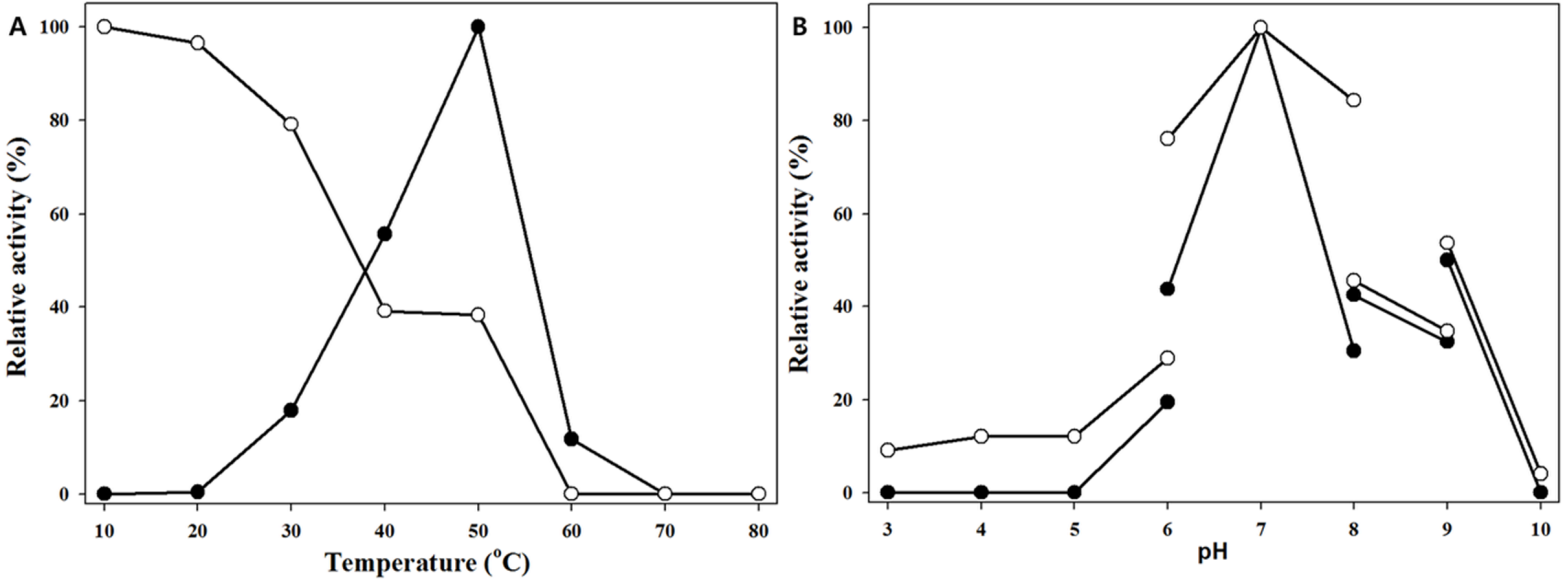
Effect of temperature and pH on MtBgl85. (A) Optimal temperature (solid circles) and thermostability (open circles) of MtBgl85. Each reaction sample was compared with the standard assay control without any additives. (B) Optimal pH (solid circles) and pH stability (open circles) of MtBgl85. Reactions were performed in the following buffers: citrate buffer for pH 3.0–6.0, sodium phosphate buffer for pH 6.0–8.0, Tris-HCl buffer for pH 8.0–9.0, and glycine-NaOH buffer for pH 9.0–10.0.

To determine the optimum temperature of MtBgl85, it was incubated at different temperatures (10–80 °C) for 15 min with *p*NP*β*G as a substrate (Fig. 4A). MtBgl85 showed maximal activity at 50 °C, approx. 50% at 40 °C, and approx. 20% at 30 °C and 60 °C, respectively. This similar result of BGL, belonging to the glycoside hydrolase family 3, has been reported, from *Fomitopsis pinicola* KMJ812 showed optimal activity at 50 °C (Joo et al., 2009).

MtBgl85 was preincubated at different temperature (10–80 °C) for 1 h to determine its thermostability. The residual activities were detected under the standard conditions. MtBgl85 was found to maintain enzyme activity under 20 °C; however, its stability was significantly reduced over 30 °C. The residual activity was 48% of maximal activity after preincubation of MtBgl85 for 1 h at 50 °C. Similarly, BGL from *Fomitopsis pinicola* KMJ812 (Joo et al., 2009) and OaBGL84 from *Olleya aquimaris* DAU311 (Hwang, Lee & Choi, 2018) showed reduced enzyme activity over 40 °C.

The effect of pH on MtBgl85 was carried out with *p*NP*β*G in a broad pH range, from 3.0 to 10.0 (Fig. 4B). MtBgl85 was active optimally at 20 mM sodium phosphate buffer (pH 7.0), 43% at pH 6.0, and 30% at pH 8.0, respectively. These results suggest that MtBgl85 has a neutral pH condition. Similarly, PxBglA from *Paenibacillus xylanilyticus* KJ-03 showed optimal activity at pH 7.0 (Park, Lee & Choi, 2013).

The pH stability of MtBgl85 remained 99% after preincubation in 20 mM sodium phosphate buffer (pH 7.0) for 1 h at 4 °C without substrate. The activity of MtBgl85 remained at 85% at pH 6.0 and 75% at pH 8.0, respectively.

### Effect of metal ions and NaCl on MtBgl85

To confirm the effect of various metal ions (Mg^2+^, Cs^2+^, Na^+^, K^+^, Ca^2+^, Li^+^, Co^2+^, Ba^2+^, Mn^2+^, Zn^2+^, Al^2+^, Sr^2+^, Cd^2+^, Cu^2+^, and Hg^2+^), MtBgl85 was pre-incubated for 1 h at 4 °C without substrate (Table 1). The final concentrations of metal ions were each 1, 5, and 10 mM, respectively. The residual activity was measured with *p*NP*β*G at 50 °C for 15 min. Most of the metal ions used in the experiments acted as enzyme inhibitors and decreased the enzyme activity by 20–80%. Hg^2+^ completely inhibited enzyme activity at all concentrations. Hg^2+^ is known to be a major inhibitor of many enzymes. It reacts with cysteine residues, specifically in –SH groups, and can change the tertiary structure of a protein (Lee, Lee & Choi, 2018). This suggested that the active site might have thiol groups that are involved in catalytic and essential for maintaining the enzyme structure. At 10 mM, Cu^2+^ and Mn^2+^ both completely inhibited enzyme activity, whereas Co^2+^ promoted enzyme activity. Similarly, BGL from *Fomitopsis pinicola* maintained 46–88% of its maximal activity in 0.1 M Cu^2+^ and Mn^2+^, respectively (Joo et al., 2009). rBGLa from *Lactobacillus antri* maintained 68–82% of its maximal activity in 0.1 M Mg^2+^ and K ^+^, and was strongly inhibited by 0.1 M Ca^2+^, respectively (Kim, Lee & Ma, 2017). PxBglA from *Paenibacillus xylanilyticus* maintained 30–70% of its maximal activity in 0.5 M Zn^2+^ and Co^2+^, and strongly inhibited by 0.5 M Cu^2+^, respectively (Park, Lee & Choi, 2013). OaBGL84 from *Olleya aquimaris* (Hwang, Lee & Choi, 2018), PxBglA from *Paenibacillus xylanilyticus* (Park, Lee & Choi, 2013) and TnBglB from *Thermotoga naphthophila* (Akram, Haq & Mukhtar, 2018) were completely inhibited by Hg^2+^.

**Table 1:** The effect of different metal ions and chemical reagents on MtBgl85.

Substances	Relative activity (%)		
	1 mM	5 mM	10 mM
Control	100	100	100
Co^2+^	67.1 ± 2.9	83 ± 12.0	128 ± 7.8
K^+^	82 ± 13.0	84 ± 2.6	86 ± 11.9
Li^+^	78 ± 3.4	74 ± 5.9	68 ± 8.0
Mg^2+^	64 ± 5.5	51 ± 3.1	42 ± 2.2
Cs^2+^	54 ± 12.1	52 ± 2.5	78 ± 8.6
Na^+^	75 ± 3.0	84 ± 11.4	71 ± 7.1
Ca^2+^	60 ± 10.0	70 ± 5.2	44 ± 4.4
Ba^2+^	62 ± 5.3	61 ± 0.7	79 ± 1.4
Zn^2+^	87 ± 4.2	45 ± 3.8	41 ± 9.2
Al^2+^	67 ± 10.9	44 ± 14.1	26 ± 5.7
Sr^2+^	80 ± 2.5	45 ± 8.9	33 ± 6.3
Cd^2+^	3 ± 5.5	13 ± 1.9	23 ± 11.1
Mn^2+^	80 ± 8.5	50 ± 11.0	N.D.
Cu^2+^	16.9 ± 2.9	3.2 ± 2.0	N.D.
Hg^2+^	N.D.	N.D.	N.D.

**Figure 5:**
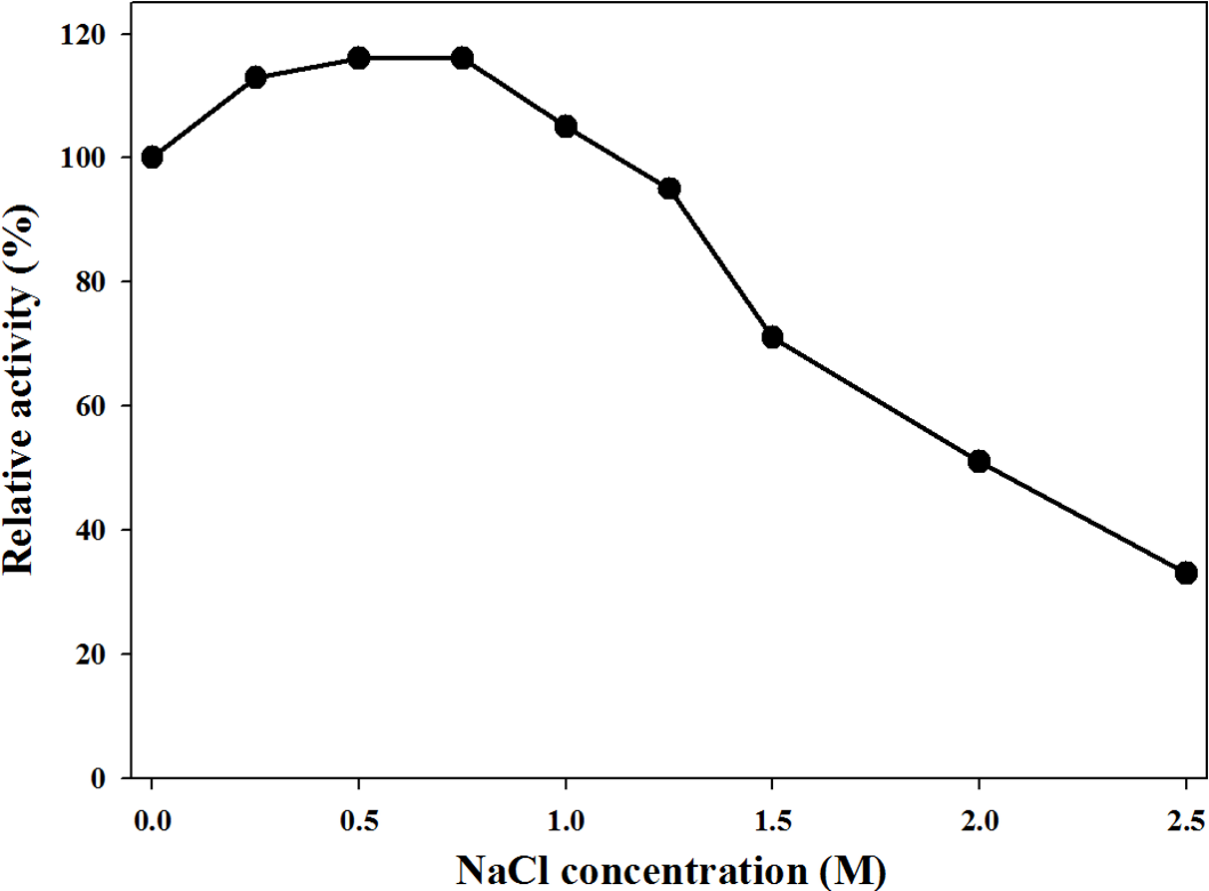
Effect of NaCl concentration on MtBgl85. MtBgl85 was preincubated on ice for 1 h at various NaCl concentrations: 0.25–2.5 M.

The effect of NaCl concentration on MtBgl85 from *M. thermotolerans* DAU221 was carried out in 20 mM phosphate buffer pH 7.0 (Fig. 5). MtBgl85 was pre-incubated with each NaCl concentration (0.25–2.5 M) at 4 °C for 1 h. Then, the residual activity was measured at 50 °C for 15 min with *p*NP*β*G. The residual activity of MtBgl85 showed the highest activity at 0.75 M NaCl, a concentration similar to the salt concentration (0.6 M) in the sea. At over 0.75 M NaCl, enzyme activity gradually decreased. MtBgl85 maintained approximately 70% of its maximal activity at 1.5 M NaCl, 50% at 2 M, and 30% at 2.5 M, respectively. Enzymes that are resistant to high salt concentrations in marine microorganisms are essential and have many industrial applications (Karthik, Binod & Pandey, 2015). Similarly, OaBGL84 from *Olleya aquimaris* maintained 94–113% of its maximal activity at 1, 5, and 10 mM NaCl, respectively (Hwang, Lee & Choi, 2018).

### Effect of various organic solvents on MtBgl85

The effect of various organic solvents (DMSO, methanol, acetonitrile, ethanol, acetone, isopropyl alcohol, butanol, isoamyl alcohol, benzene, toluene, and hexane) on the MtBgl85 were confirmed (Table 2). Organic solvents were added into a standard assay mixture and incubated for 15 min at 50 °C. MtBgl85 showed higher enzyme stability at non-polar solvents (high Log *P*_*ow*_) than polar solvents (low Log *P*_*ow*_), respectively. In polar solvents (low Log *P*_*ow*_), MtBgl85 maintained higher enzyme stability at low concentrations (10%, v/v) than at high concentration (30% or 50%, v/v). In non-polar solvents (high Log *P*_*ow*_), MtBgl85 maintained higher enzyme stability at high concentrations (50%, v/v) than at low concentrations (10% or 30%, v/v) (Table 2). Similarly, OaBGL84 from *Olleya aquimaris* showed higher stability at non-polar solvents (high Log *P*_*ow*_) than at polar solvents (low Log *P*_*ow*_) (Hwang, Lee & Choi, 2018). TnBglB from *Thermotoga naphthophila* maintained higher stability at low concentrations (10%, v/v) than at high concentrations (30% or 50%, v/v) in ethanol, methanol, isopropyl alcohol, and butanol, respectively (Akram, Haq & Mukhtar, 2018). Although there was no clear relationship between the stability of enzyme and solvent polarity, non-polar solvents may do not compete for the hydration shell around the enzyme (Batra & Mishra, 2013; Chamoli et al., 2016). These organic solvent tolerance *β*-glucosidases have competence to work in presence of organic solvents that can look for wide application in various industries such as paper, pulp, and textile industries and other biomass conversion processes (Chamoli et al., 2016).

**Table 2:** The effect of various organic solvents on MtBgl85.

Organic solvents	Log *P*_*ow*_	Relative activity (%)		
		10% (v/v)	30% (v/v)	50% (v/v)
Control		100	100	100
DMSO	−1.35	79 ± 0.7	29 ± 2.3	N.D.
Methanol	−0.76	124 ± 5.3	16 ± 11.7	2 ± 5.2
Acetonitrile	−0.34	20 ± 3.5	N.D.	N.D.
Ethanol	−0.24	85 ± 4.5	15 ± 3.0	1 ± 3.4
Acetone	−0.24	93 ± 2.8	12 ± 2.7	N.D.
Isopropyl alcohol	0.16	78 ± 3.2	22 ± 7.2	1 ± 1.0
Butanol	0.80	33 ± 4.9	48 ± 14.5	99 ± 6.8
Isoamyl alcohol	1.28	97 ± 0.7	85 ± 4.8	139 ± 6.2
Benzene	2.13	68 ± 7.8	72 ± 24.7	116 ± 26.7
Toluene	2.40	87 ± 31.0	115 ± 9.2	103 ± 17.4
Hexane	3.50	103 ± 0.1	99 ± 4.5	88 ± 2.4

**Notes.**

± standard error N.D not detected

### Substrate specificity and enzyme kinetics

The activity of MtBgl85 with various substrates was determined (Table S1). The highest activity of MtBgl85 was observed with *p*NP*β*G as a substrate. Among the *p*-nitrophenyl derivatives, MtBgl85 showed 2% of its maximal activity toward *p*NP*α*G, but no activity toward *p*NP*β*C, *p*NP*β*X, *p*NP*β*Gal, and *p*NP*α*R. Among the saccharides, MtBgl85 hydrolyzed esculin and arbutin, but could not hydrolyze avicel, cellobiose, maltose, lactose, sucrose, or xylan. BaBgl3 from *Bifidobacterium adolescentis* showed maximal activity using *p*NP*β*G as a substrate and considerable activity on *p*NP *β*X, amounting to almost 32% of its activity on *p*NP*β*G (Florindo et al., 2018). Similarly, BGL1 from *Thermoascus aurantiacus* hydrylozed artificial substrates such as *p*NP*β*G and *p*NP*β*C, as well as cellobiose and cello-oligosaccharides (Hong, Tamaki & Kumagai, 2007). TnBglB from *Thermotoga naphthophila* exhibited maximal activity towards *p*NP*β*G and could hydrolyze *p*NP*β*X, *p*NP*β*C, *p*NP*β*Gal, and cellobiose (Akram, Haq & Mukhtar, 2018). On the basis of substrate specificity, *β*-glucosidases have been classified as (1) aryl *β*-glucosidases, which act on aryl-glucosides, (2) true cellobiases, which hydrolyze cellobiose to release glucose, and (3) broad substrate specificity enzymes, which act on a wide spectrum of substrates (Bhatia, Mishra & Bisaria, 2002). These results demonstrate that MtBgl85 has aryl *β*-glucosidase.

The kinetic constant of MtBgl85 was determined by using the Lineweaver-Bulk plot with different concentrations of *p*NP*β*G as a substrate. The *K*_*m*_ and *V*_*max*_ values were 0.29 mM and 0.354 U/mg, respectively (Fig. S3).

### Effect of glucose on MtBgl85

**Figure 6:**
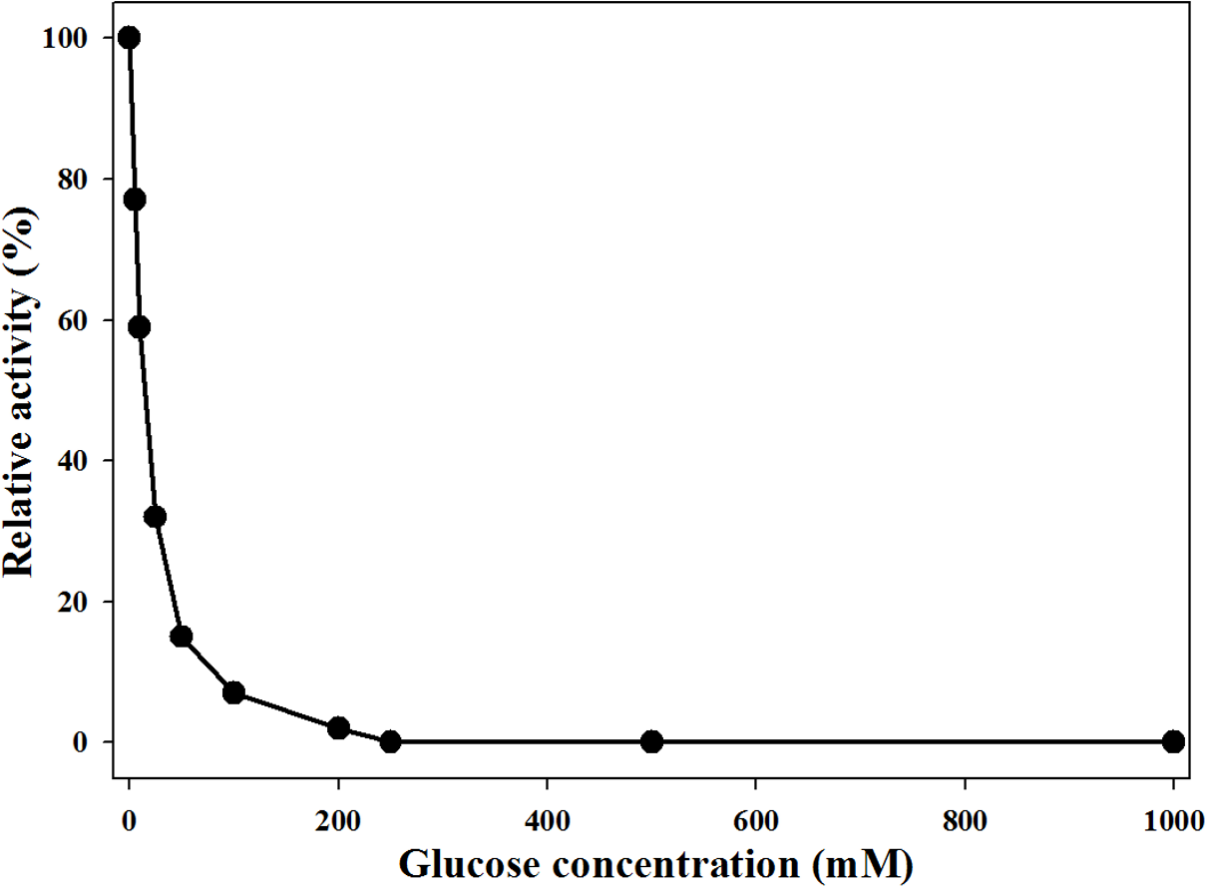
Effect of glucose concentration on MtBgl85.

Competitive inhibition by glucose is a common characteristic of *β*-glucosidases that limits their use in enzymatic hydrolysis (Li et al., 2012). Glucose inhibition of MtBgl85 was investigated using various concentrations of glucose (0–1,000 mM). The residual activity of MtBgl85 was drastically reduced to 59% and 7% of its initial activity in the presence of 10 mM and 100 mM glucose, respectively (Fig. 6). Similarly, the residual activity of TnBglB from *Thermotoga naphthophila* was drastically reduced to 12% and 18% in the presence of 600 mM glucose (Akram, Haq & Mukhtar, 2018). BaBgl3 from *Bifidobacterium adolescentis* was significantly inhibited by adding glucose, since it was shown to be a competitive inhibitor for BaBgl3 (Florindo et al., 2018). OaBGL84 retained 40% and 17% of its initial activity, in 100 and 200 mM glucose, respectively (Hwang, Lee & Choi, 2018). Many *β*-glucosidases, including MtBgl85, are sensitive to glucose, hence they are easily inhibited by the end product feedback (glucose).

### HPLC analysis of hydrolysis products by MtBgl85

**Figure 7:**
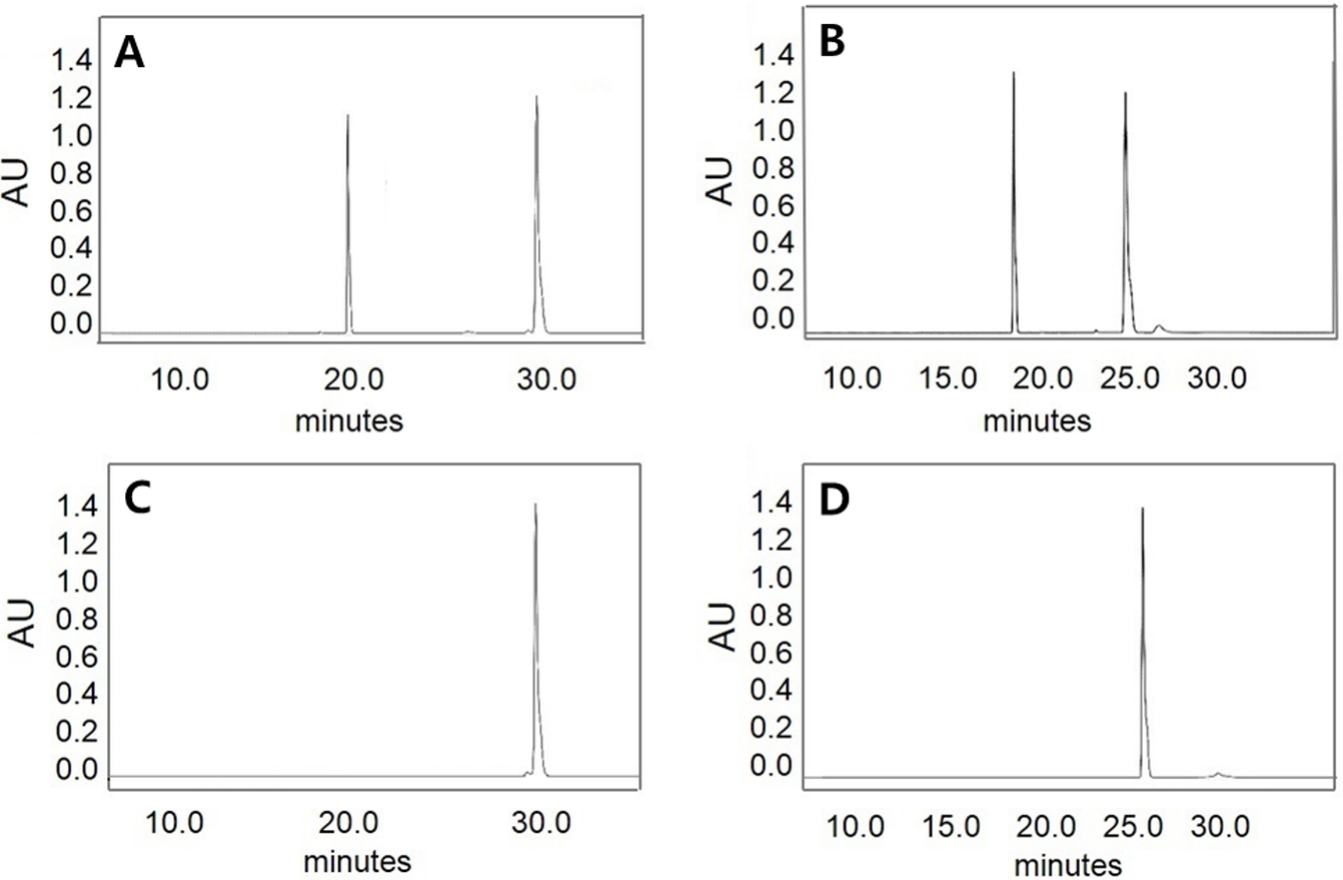
HPLC analysis of isoflavone hydrolysis products by MtBgl85. (A) Standard of genistin (20.5 min) and genestein (31 min); (B) Standard of daidzin (17.9 min) and daidzein (25 min); (C) Hydrolysis product of genisitin by MtBgl85; (D) Hydrolysis product of daidzin by MtBgl85. Isoflavone glycosides were incubated with MtBgl85 at 50 °C for 15 min.

**Figure 8:**
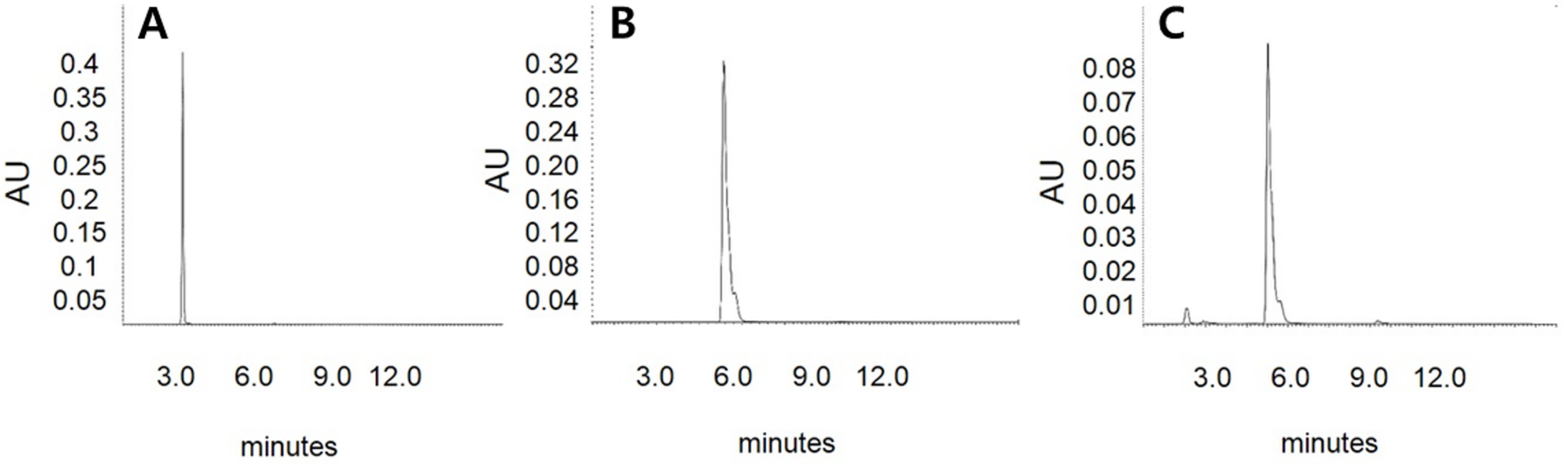
HPLC analysis of arbutin hydrolysis products by MtBgl85. (A) Standard of arbutin (3 min); (B) Standard of hydroquinone (6 min); (C) Hydrolysis products of arbutin by MtBgl85. Arbutin was incubated with MtBgl85 at 50 °C for 30 min.

The reaction products of MtBgl85 were examined using isoflavone glycosides (daidzin or genistin) or arbutin as a substrate at 50 °C and analyzed by HPLC (Fig. 7). Isoflavone glycosides (genistin and daidzin) were completely converted to isoflavone aglycones (genistein and daidzein) by MtBgl85. Similarly, PxBglA from *Paenibacillus xylanilyticus* (Park, Lee & Choi, 2013) and OaBGL84 from *Olleya aquimaris* (Hwang, Lee & Choi, 2018) converted isoflavone glycons (genistin and daidzin) into isoflavone aglycones (genistein and daidzein), respectively. Likewise, arbutin was hydrolyzed to hydroquinone and glucose by MtBgl85 (Fig. 8). PxBglA from *Paenibacillus xylanilyticus* used arbutin as a substrate (Park, Lee & Choi, 2013). Further work to improve isoflavone glycoside transformation via the structural modification of the enzymes by molecular modeling on the basis of the solved three-dimensional structures is in progress.

## Conculsion

In the post-genome period, the enzyme identification from the whole genome sequence still depends excessively on biochemical characterization of the novel enzyme. Novel enzymes can be usefully applied in various industrial applications. A potent isoflavone converting enzyme, MtBgl85, was identified from *Microbulbifer thermotolerans* DAU221. MtBgl85 belongs to glycoside hydrolase family 3 and showed high activity toward *p*NP*β*G as a substrate at 50 °C and pH 7.0. MtBgl85 also maintained the enzyme activity in the presence of high-salt conditions and non-polar solvents (high Log *P*_*ow*_). These results suggest MtBgl85 is an influential candidate for the biodegradation process and can also be efficiently used in beverage, cosmetic, feed, food, pharmaceutical and paper industries.

##  Supplemental Information

10.7717/peerj.7106/supp-1 Data S1 Raw data for Figs. 4–6 Fig. 4: raw data of the optimal temperature and pH and temperature and pH stability for MtBgl85 of M. thermotolerans DAU221.
Fig. 5: raw data of the NaCl effect on MtBgl85 of M. thermotolerans DAU221.
Fig. 6: raw data of the glucose effect on MtBgl85 of M. thermotolerans DAU221.

10.7717/peerj.7106/supp-2 Figure S1 Nucleotide sequences and deduced amino acid sequences of MtBgl85 from *M. thermotolerans* DAU221 This has been deposited in GenBank under the accession number MK408673.

10.7717/peerj.7106/supp-3 Figure S2 Unrooted neighbor-joining phylogenetic tree of MtBgl85 and *β*-glucosidases The amino acid sequences of the bacterial * β*-glucosidases were referred to the glycoside hydrolase family 1 and 3. Sequence alignment was performed using ClustalW and the tree was created with the MEGA program version 7. The scale bar represents the number of substitutions per site.

10.7717/peerj.7106/supp-4 Figure S3 Kinetic studies for MtBgl85 Kinetic studies for MtBgl85 catalyzed hydrolysis at various final concentrations (0.02-0.1 mM) of *p*NP*β*G as the substrate based on the Lineweaver-Burk plot.

10.7717/peerj.7106/supp-5 Table S1 Substrate specificity of MtBgl85 from *M. thermotolerans* DAU221 Each reaction sample was compared with the standard assay control.

## Additional Information and Declarations

### Data Availability

The following information was supplied regarding data availability: The nucleotide sequence is available in GenBank: MK408673/CP014864.
