# Supplementary material for: Cloning, purification, and characterization of GH3 β-glucosidase, MtBgl85, from Microbulbifer thermotolerans DAU221
Source: PeerJ. 2019 Jul 22;7:e7106. doi: 10.7717/peerj.7106 (PMC6657685; doi:10.7717/peerj.7106)
Supplement: Figure S1 — This has been deposited in GenBank under the accession number MK408673. [file peerj-07-7106-s002.pdf]

ATGAAAAAGTATCCCATTTATATTGCCGCTATGGCCGGGCTTCTCTCGCTCGCCGCCACT 60
M K K Y P I Y I A A M A G L L S L A A T
CTGCCACTTCAGGCCGCCAACGAATCTGTGGCTACCACCCAATCCCACAGCCAACAAAAT 120
L P L Q A A N E S V A T T Q S H S Q Q N
GCCATCGAGTCCCGTATCCAGAGCCTACTCGCACAAATGTCCGTGCAGGAAAAAATCGGT 180
A I E S R I Q S L L A Q M S V Q E K I G
CAGCTGGCACTGCGCGACTGGGGAACCTTCAAGGCCGAGGATATGAAAGATATCAAGCAG 240
Q L A L R D W G T F K A E D M K D I K Q
GCAATACGCGAAGGACGCGTCGGAGGGTTCCTGAATGTCAACTTCAGTTCCGTGGATAAC 300
A I R E G R V G G F L N V N F S S V D N
AATGCATTCGAGGAATTGCAACGCCTCGCCGTGGAAGAAAGCCCCCGGGGCATTCCGCTT 360
N A F E E L Q R L A V E E S P R G I P L
CTGTTTGGGCAGGATGTAATCCACGGCTACGAAACCATTTTCCCAATTCCTCTCGGCCAA 420
L F G Q D V I H G Y E T I F P I P L G Q
GCCGCCAGCTGGAATCCGGAACTGATTAGAAACGGGGCGCGCATCGCCGCACAAGAGGCC 480
A A S W N P E L I R N G A R I A A Q E A
AGCGCCGATGGCATCCGCTGGACCTTTGCACCCATGATCGACATCAGCCGCGACCCGCGC 540
S A D G I R W T F A P M I D I S R D P R
TGGGGCCGCATCGCCGAAACCCTGGGGGAAGATCCTCTGCTGACCTCGATCCTCGGTGTC 600
W G R I A E T L G E D P L L T S I L G V
GCCATGGTGGAAGGTTTCCAGACTGACAACCTCTCCGATCCCACCAGCCTCGCTGCCTGT 660
A M V E G F Q T D N L S D P T S L A A C
GGCAAACACTTTGCCGGCTACGGTGCCGCAGAAGGCGGCCGCGATTACAACAGTGCTTAT 720
G K H F A G Y G A A E G G R D Y N S A Y
ATTCCCGAGCGCCTGCTACGCGATATCTACCTGCCGCCGTTCAAGGCCGGTATAGACGCA 780
I P E R L L R D I Y L P P F K A G I D A
GGTATGCAGAGCATCATGAGTACCTACAGCTCCCTCAACGATGTCCCCGGCACTGGTAGC 840
G M Q S I M S T Y S S L N D V P G T G S
CCCTTCCTGTTCAAACAGATTCTGCGAGACGAATGGGGGTTTGACGGCTTTGTAGTAAGT 900
P F L F K Q I L R D E W G F D G F V V S
GACTGGAACGCGGTGATGGAAATGATTCCTCACGGCTATGCTCGCGACGCCAAGCACGCC 960
D W N A V M E M I P H G Y A R D A K H A
GCCGCCCTTGCCGCCAATGCGGGCATCGATATGGAGATGCACACGGACACCTACGAACAA 1020
A A L A A N A G I D M E M H T D T Y E Q
TTCCTGGCAAGATTAATGGACGAAGGGGAATTTACCGAAGCACAGTTAGATACAGCGGTG 1080
F L A R L M D E G E F T E A Q L D T A V
GCCAATATCCTGCGGGTAAAACTGCGCTTAGGACTGTGGGAAAACCCCTACCCCCGAGCA 1140
A N I L R V K L R L G L W E N P Y P R A
AAAACGCCAGCCGAGCGTGAGCAGATCATCCGCAACGACCAATTCCTGAATGCCGCCAAA 1200
K T P A E R E Q I I R N D Q F L N A A K
GAGGCCGCCAAGGAAACCTTCGTCCTACTCAAAAACGACAAACAGCTGCTGCCGCTGAAG 1260
E A A K E T F V L L K N D K Q L L P L K
AAAGGCCAGACGGTCGCACTTATTGGCCCATTGGCGGATGCCCCTCACGAGCAGATGGGC 1320
K G Q T V A L I G P L A D A P H E Q M G
ACTTGGGTGTACAACGGTAACAAAGAACACTCGCGCACCCTGCTACCTGCACTGCGCGCG 1380
T W V Y N G N K E H S R T L L P A L R A

ATGGTGGGTGATAAAGGTAAAATTCTCTACGCCGCCGGCCTCGAATACAGCCGGGACACC 1440
M V G D K G K I L Y A A G L E Y S R D T
AGCACGCGCGGTTTCAAAGCGGCAATCAAAGCGGCCAAAAAAGCCGATGTAGTGCTTTTT 1500
S T R G F K A A I K A A K K A D V V L F
GTCGGCGGCGAGGAAGCTATCCTCTCCGGCGAGGGCCACAGCCGCGGCGATATTCGCCTG 1560
V G G E E A I L S G E G H S R G D I R L
CCCGGCGCACAGGCGCAACTGCTGGCAGAGCTGGCCAAGACCGGCAAGCCGCTGGCAATG 1620
P G A Q A Q L L A E L A K T G K P L A M
GTATTGATGGCTGGGCGCCCGTTGCAGCTGGATCAAACCCTCGAACAGGCCGACGCAGTG 1680
V L M A G R P L Q L D Q T L E Q A D A V
ATGATGGCCTGGCACCCGGGAACCATGGCCGGACCGGCACTGGCGGAGGTGCTCTACGGA 1740
M M A W H P G T M A G P A L A E V L Y G
GAAACCTCACCTTCCGGCCGCCTGCCGTTGAGCTGGCCGGTGGGCGCGGGCCAGATCCCT 1800
E T S P S G R L P L S W P V G A G Q I P
ATCTATTACAACCACCTACCTACTGGCCGTCCGGCAACCGATGACAATTACACTCGTATC 1860
I Y Y N H L P T G R P A T D D N Y T R I
GAAAAAATCGGTCGCGAGGTGTTTCAGCATCAGCCGGGCAACTCATCCAATCTGCTGGAT 1920
E K I G R E V F Q H Q P G N S S N L L D
TACGGGCACAAACCGCTGTTCCCATTCGGTTACGGCCTCACCTACAGCGAATTCCATTAC 1980
Y G H K P L F P F G Y G L T Y S E F H Y
AGTGAACTGGAACTTTCCGACAGCACTCTGGATGCAGATGGACAATTGCATGTTTCCGCC 2040
S E L E L S D S T L D A D G Q L H V S A
ACCATCACCAACACCGGCAAGCGCACCGCCACCGAAACCGTGCAATTGTATGTGCGCGAC 2100
T I T N T G K R T A T E T V Q L Y V R D
TTGGTGGGCAGTGTGTCGCGGCCGGTACGGGAATTAAAGGGTTTCGAGCGTATTACCCTG 2160
L V G S V S R P V R E L K G F E R I T L
GCGCCCGGAGAATCCCGTCGCGTGAGTTTTACACTTAAAGCCAACCAGCTGGCCTTCCAC 2220
A P G E S R R V S F T L K A N Q L A F H
AACGCGCAGATGCAACAGGTGATCGAGCCCGGTGAATTCCATGTATGGATTGCACCAGAC 2280
N A Q M Q Q V I E P G E F H V W I A P D
GCAGAATCGGGCTTGCAGGGCAGCTTTACCCTGCAATAA 2319
A E S G L Q G S F T L Q *
