# Supplementary figures and images for: Cloning, purification, and characterization of GH3 β-glucosidase, MtBgl85, from Microbulbifer thermotolerans DAU221
Source: PeerJ. 2019 Jul 22;7:e7106. doi: 10.7717/peerj.7106 (PMC6657685; doi:10.7717/peerj.7106)

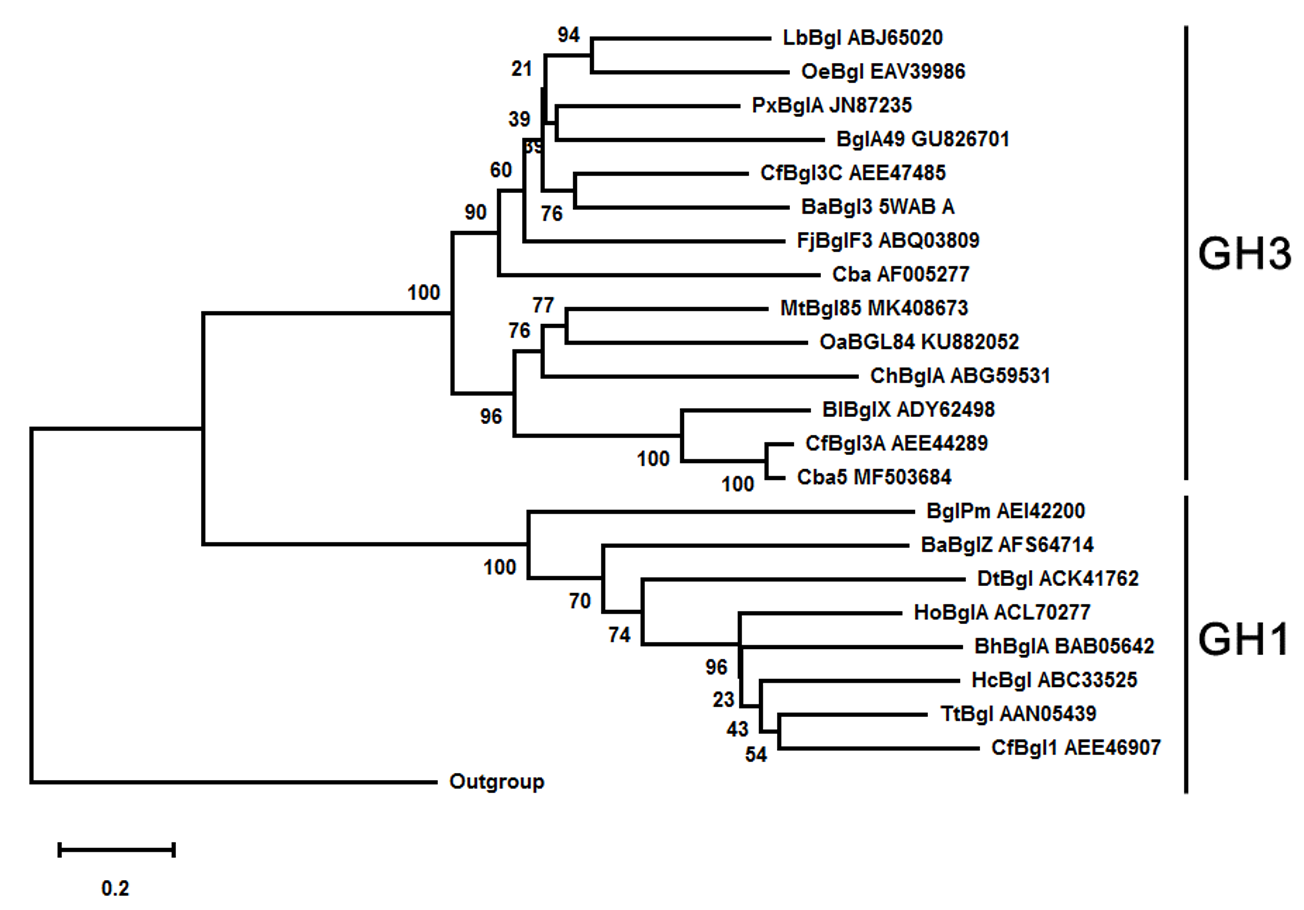

Supplement: Figure S2 — The amino acid sequences of the bacterial β-glucosidases were referred to the glycoside hydrolase family 1 and 3. Sequence alignment was performed using ClustalW and the tree was created with the MEGA program version 7. The scale bar represents the number of substitutions per site. [file peerj-07-7106-s003.png]

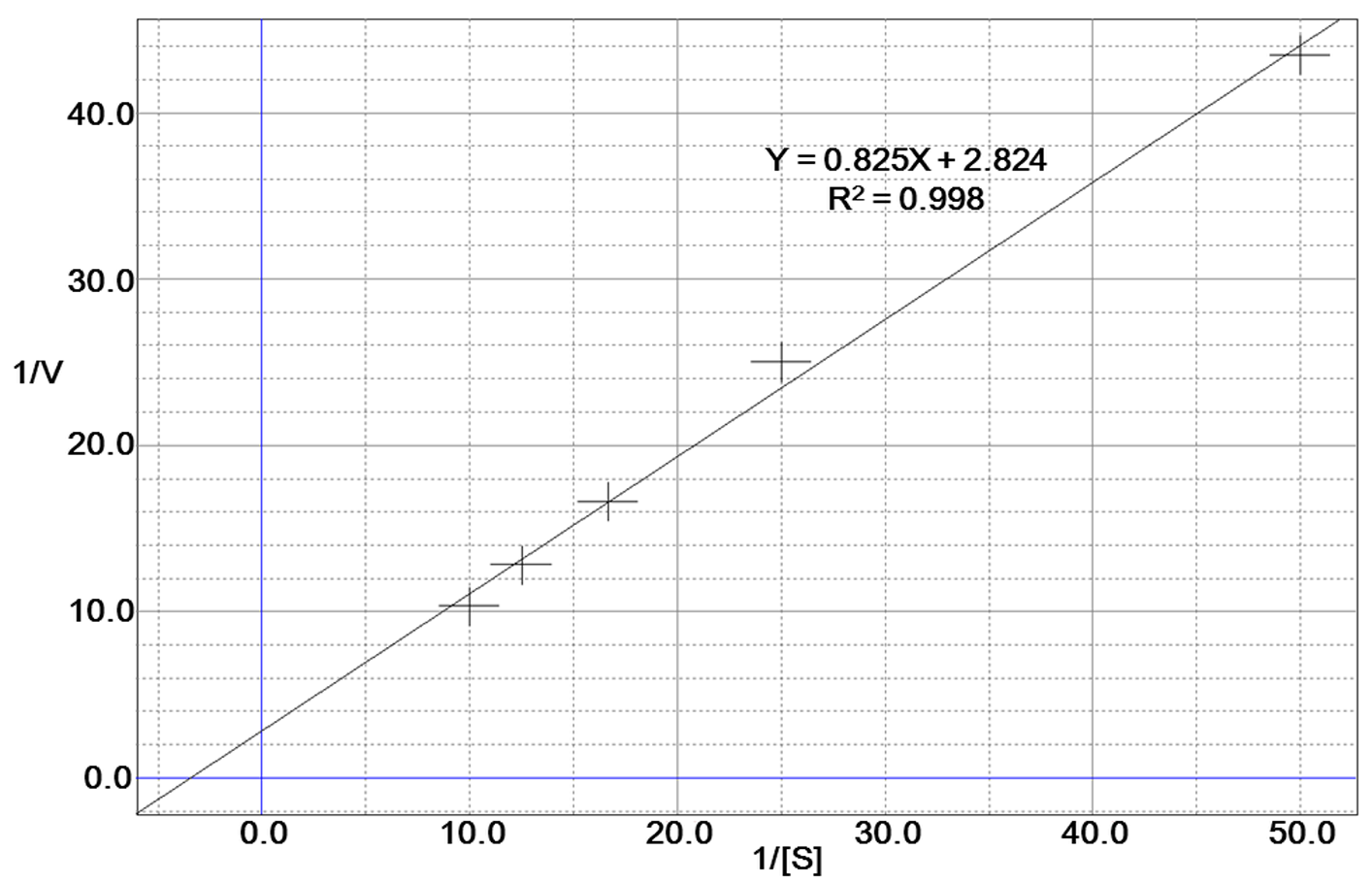

Supplement: Figure S3 — Kinetic studies for MtBgl85 catalyzed hydrolysis at various final concentrations (0.02-0.1 mM) of pNPβG as the substrate based on the Lineweaver-Burk plot. [file peerj-07-7106-s004.png]
