# Supplementary material for: Cloning, purification, and characterization of GH3 β-glucosidase, MtBgl85, from Microbulbifer thermotolerans DAU221
Source: PeerJ. 2019 Jul 22;7:e7106. doi: 10.7717/peerj.7106 (PMC6657685; doi:10.7717/peerj.7106)
Supplement: Table S1 — Each reaction sample was compared with the standard assay control. [file peerj-07-7106-s005.docx]

| Substrates | Relative activity (%) |
| --- | --- |
| *p*NP*β*G | 100 |
| *p*NP*α*G | 2 ± 0.5 |
| *p*NP*β*C | N.D. |
| *p*NP*β*X | N.D. |
| *p*NP*β*Gal | N.D. |
| *p*NP*α*R | N.D. |

N.D., not detected; ±, standard error
